# Impact of physical activity on symptoms of depression and anxiety in adolescents during the COVID-19 pandemic

**DOI:** 10.3389/fpsyt.2025.1631978

**Published:** 2025-08-25

**Authors:** Hai-Ying Yang, Li-Hong Sun

**Affiliations:** ^1^ School of Physical Education, Southwest Jiaotong University, Chengdu, Sichuan, China; ^2^ School of Physical Education, Hubei Second Normal University, Wuhan, Hubei, China

**Keywords:** adolescents, physical activity, depression, anxiety, COVID-19, mental health, risk factors

## Abstract

**Background:**

Adolescents faced increased psychological distress during the COVID-19 pandemic. While prior research suggests physical activity (PA) may mitigate depression and anxiety, findings have been inconsistent and rarely focus on adolescents during prolonged lockdowns. This study addresses this gap by evaluating the association between daily PA duration and mental health outcomes among Chinese adolescents during the pandemic.

**Methods:**

A cross-sectional online survey was conducted from June 1–30, 2020, among 1,142 adolescents aged 11–18 years in Pidu District, Chengdu City, China. Depressive and anxiety symptoms were assessed using the Center for Epidemiological Studies Depression Scale for Children (CES-DC) and the Generalized Anxiety Disorder 7-item scale (GAD-7), respectively. PA was self-reported and categorized as <30, 30–60, and >60 minutes/day. Logistic regression was used to estimate odds ratios (ORs) for depression (CES-DC >15) and anxiety (GAD-7 ≥5), using ≥30 min/day as the reference.

**Results:**

Depressive symptoms were reported by 40.7% of participants and anxiety symptoms by 24.1%. Compared to adolescents with ≥30 min/day of PA, those with <30 min/day had significantly higher odds of depression (OR = 1.722, 95% CI: 1.342–2.226) and anxiety (OR = 1.653, 95% CI: 1.299–2.521). Additional independent predictors included female sex, sleep duration <6 hours, and self-reported decline in learning efficiency.

**Conclusions:**

Insufficient PA (<30 min/day) was independently associated with elevated depression and anxiety symptoms in adolescents during the pandemic. These findings support promoting ≥30 minutes of daily PA as a scalable, non-pharmacological strategy to protect adolescent mental health during public health emergencies.

## 1 Introduction

Adolescents have been particularly vulnerable to the psychological impacts of the COVID-19 pandemic, with significant increases in symptoms of depression and anxiety (1, 2). While physical activity (PA) has long been proposed as a protective factor against mental health issues, the pandemic has drastically altered the types and amounts of PA that are feasible for adolescents (3, 4). Before the pandemic, common forms of PA such as team sports, physical education, and outdoor group activities played a major role in adolescent well-being (5, 6). However, during the pandemic, restrictions limited access to gyms, sports teams, and recreational spaces, making home-based activities like bodyweight exercises, yoga, and walking crucial alternatives (7–9). These changes may have influenced the overall duration and intensity of PA, potentially altering its effects on mental health.

Recent literature underscores the mental health benefits of physical activity (PA), particularly for adolescents experiencing elevated psychological distress during the COVID-19 pandemic. Systematic reviews by Li et al. (10) and Wolf et al. (11) demonstrate that higher levels of moderate-to-vigorous PA are associated with lower rates of depression and anxiety. Samji et al. (12) further identify adolescents—especially females and older youth—as disproportionately affected by pandemic-related disruptions, highlighting PA as a potential protective factor. While intervention studies such as Erdoğan et al. (13) suggest that structured PA (e.g., yoga) may improve psychological outcomes in subgroups like obese adolescents, their findings are limited in scope and generalizability. However, several critical gaps remain. Existing studies often aggregate broad age ranges, obscuring insights specific to adolescents. Most do not differentiate between varying durations of PA or establish clear dose-response thresholds under conditions of social isolation. Additionally, few studies have examined these associations during prolonged lockdown periods, when routine PA may be severely constrained and mental health impacts intensified.

The present study addresses these gaps by focusing on a large cohort of Chinese adolescents during the early COVID-19 lockdown. By stratifying daily PA into three distinct categories and analyzing its association with depression and anxiety symptoms—while adjusting for key confounders such as gender, sleep duration, and learning efficiency—this study offers new evidence on the dose-dependent protective effects of PA. These findings contribute to a more nuanced understanding of how daily physical activity may buffer adolescent mental health during global crises.

## 2 Methods

### 2.1 Study design

The data for this study were collected from a cross-sectional online survey conducted between June 1, 2020, and June 30, 2020, using the “Wenjuanxing” platform via WeChat. The participants were adolescents aged 11 to 18 years who were enrolled in middle or high school in our city and resided there during the COVID-19 pandemic. Each mobile phone number was permitted to participate only once in the survey. A total of 1142 middle and high school students were included in the analysis. The age range of the participants was 11 to 18 years, with a mean age of 15.3 ± 1.8 years. Prior to participation, all participants and their guardians electronically signed an informed consent form. This study was conducted in strict accordance with the ethical principles outlined in the Declaration of Helsinki and was approved by the institutional review board of our institution. The questionnaire did not collect names or other personally identifiable information and was fully anonymous. The questionnaire was anonymous, and no personally identifiable information was collected. All data were used solely for research purposes by the study team.

### 2.2 Survey methodology and content

his study employed an online survey to collect data on the demographic characteristics, clinical features, and lifestyle habits of adolescents, as well as to assess their levels of depression and anxiety during the COVID-19 pandemic. The survey consisted of several key sections designed to collect comprehensive data on the study participants.

1. Demographic Information: This section gathered basic demographic details, including the participant’s age, gender, residential area, grade level, and school type.
2. Study and Lifestyle Habits: The survey examined participants’ daily habits over the past week, including average sleep duration, study time, changes in study efficiency, and the time spent engaging in physical activity each day. PA was assessed with a single self-report item adapted from descriptors used in the International Physical Activity Questionnaire–Short Form (IPAQ−SF). Participants were asked: “During the past 7 days, on average how many minutes per day did you spend doing moderate−to−vigorous physical activities (e.g., brisk walking, running, cycling, ball games, or other sports) that made you breathe somewhat harder or much harder than normal?” Respondents entered a numeric value (minutes/day). To anchor intensity, examples were provided and light activities (e.g., casual walking, household chores) were explicitly excluded. Based on *a priori* cut−points, daily PA was categorized as <30 min, 30–60 min, and >60 min. These categories were selected to distinguish clearly insufficient PA (<30 min/day), sub−recommended levels (30–60 min/day), and meeting/exceeding the widely promoted target of ≥60 min/day for adolescents.
3. Depression Assessment: The Center for Epidemiological Studies Depression Scale for Children (CES-DC) was used to assess depressive symptoms in adolescents aged 6 to 17 years. The CES-DC is a brief, structured self-assessment tool designed to measure depressive symptoms experienced in the past week. It includes 20 items across four dimensions: depressive mood, positive mood (reverse scored), somatic symptoms, and interpersonal relationships. Each item is scored on a 0–3 scale, with 0 indicating “not at all,” 1 indicating “a little,” 2 indicating “sometimes,” and 3 indicating “most of the time.” The total score ranges from 0 to 60, with a score above 15 indicating the presence of depressive symptoms, and a score of 20 or greater suggesting significant depressive symptoms.
4. Anxiety Assessment: The Generalized Anxiety Disorder 7-Item Scale (GAD-7) was employed to assess anxiety symptoms in participants over the past two weeks. The GAD-7 is a concise self-report tool consisting of 7 items that evaluate the frequency of anxiety symptoms. A 4-point scoring system is used: 0 for “not at all,” 1 for “several days,” 2 for “more than half the days,” and 3 for “nearly every day.” A total score of 5 or higher suggests the presence of anxiety symptoms. Higher scores indicate more severe anxiety, with thresholds of 5, 10, and 15 denoting mild, moderate, and severe anxiety, respectively.

### 2.3 Quality control

To ensure the accuracy and reliability of the data collected, several quality control measures were implemented throughout the study process. The survey was administered via the “Wenjuanxing” platform, which provided a secure, user-friendly interface for respondents. The following quality control strategies were applied:

1. Exclusion of Inconsistent Data: To ensure data validity, responses containing obvious logical inconsistencies were excluded. This included participants whose answers to certain questions were contradictory or those who completed the survey in an abnormally short amount of time. Specifically, any survey with a completion time of less than 120 seconds was removed, as this suggested insufficient attention to the questions.
2. One Response Per Participant: Each mobile phone number was permitted to participate only once in the survey. This helped to prevent duplicate entries and ensured that the sample was representative of individual adolescents rather than repeated submissions from the same participants.
3. Age Range Verification: Only adolescents aged 11 to 18 years were included in the study. Responses from individuals outside of this age range were excluded to maintain the focus of the study on the target population. This age filter was strictly enforced during the data cleaning process.
4. Data Monitoring: Regular monitoring of survey responses was conducted throughout the data collection period to identify any potential issues with the survey or platform. This allowed for real-time corrections and ensured that data collection proceeded smoothly.

### 2.4 Statistical analysis

Data were analyzed using SPSS version 28.0. The data were initially categorized into quantitative and categorical variables, and normality tests were conducted to assess the distribution of quantitative data. For normally distributed quantitative data, inter-group comparisons were performed using independent sample t-tests, and the results were reported as mean ± standard deviation (SD). Categorical variables were presented as frequencies and percentages. The Chi-square (χ²) test was used to examine associations or independence between categorical variables. In cases where the assumptions for the Chi-square test were not met, Fisher’s exact test was employed as an alternative. Multivariate associations were explored using logistic regression analysis to identify factors associated with depressive or anxiety symptoms. For descriptive analyses, PA was treated as a three−level categorical variable (<30, 30–60, >60 min/day). For multivariable logistic regression, PA was dichotomized as <30 min/day versus ≥30 min/day to improve model stability and interpretability; the ≥30 min/day group served as the reference. All statistical tests were two-tailed, and a p-value of less than 0.05 was considered statistically significant.

## 3 Results

### 3.1 Demographic and clinical characteristics

Among the 1,142 adolescents included in the analysis, 465 participants (40.7%) had CES-DC scores >15, indicating depressive symptoms, while 357 (31.3%) scored ≥20, suggesting more severe depressive states. A total of 275 participants (24.1%) met the threshold for anxiety symptoms, defined as GAD-7 scores ≥5. Comparative analyses revealed several significant associations between demographic/clinical characteristics and the presence of depression and anxiety. Higher symptom prevalence was observed among older adolescents (15–18 years), females, and senior high school students (all P < 0.05). In contrast, school type and adherence to COVID-19 protective measures were not significantly associated with mental health status. Regarding behavioral factors, short sleep duration (<6 hours per night) was strongly associated with both depressive and anxiety symptoms (P < 0.001 for both). Adolescents reporting decreased learning efficiency also showed significantly higher rates of depression and anxiety compared to their peers (P < 0.001). Similarly, daily physical activity of less than 30 minutes was significantly associated with increased prevalence of both depression (P < 0.001) and anxiety (P = 0.009). No significant associations were found between daily study time and either mental health outcome (P > 0.05) (**Table 1**).

**Table 1:** Demographic and clinical characteristics of the study participants with depression and anxiety status.

Variables	Depression (Yes)	Depression (No)	P	Anxiety (Yes)	Anxiety (No)	P
Age (years)			0.001			0.027
11-14	74 (31.2%)	163 (68.8%)		44 (18.6%)	193 (81.4%)	
15-18	391 (43.2%)	514 (56.8%)		231 (25.5%)	674 (74.5%)	
Gender			0.048			0.007
Male	163 (37.0%)	277 (63.0%)		87 (19.8%)	353 (80.2%)	
Female	302 (43.0%)	400 (57.0%)		188 (26.8%)	514 (73.2%)	
Grade			<0.001			0.030
Junior secondary school	102 (32.3%)	214 (67.7%)		62 (19.6%)	254 (80.4%)	
Senior high school	363 (43.9%)	463 (56.1%)		213 (25.8%)	613 (74.2%)	
School type			0.153			0.460
Key school	97 (36.9%)	166 (63.1%)		68 (25.9%)	195 (74.1%)	
Non-key school	368 (41.9%)	511 (58.1%)		207 (23.5%)	672 (76.5%)	
Protection measures			0.616			0.108
Yes	461 (40.6%)	674 (59.4%)		271 (23.9%)	864 (76.1%)	
No	4 (57.1%)	3 (42.9%)		4 (57.1%)	3 (42.9%)	
Daily sleep duration (h)			<0.001			<0.001
<6	42 (73.7%)	15 (26.3%)		29 (50.9%)	28 (49.1%)	
6–8	295 (41.3%)	419 (58.7%)		166 (23.2%)	548 (76.8%)	
>8	128 (34.5%)	243 (65.5%)		80 (21.6%)	291 (78.4%)	
Daily study time (h)			0.418			0.587
≤4	344 (41.5%)	485 (58.5%)		196 (23.6%)	633 (76.4%)	
>4	121 (38.7%)	192 (61.3%)		79 (25.2%)	234 (74.8%)	
Learning efficiency			<0.001			<0.001
Lower than before	191 (57.5%)	141 (42.5%)		114 (34.3%)	218 (65.7%)	
The same as before	220 (33.6%)	435 (66.4%)		120 (18.3%)	535 (81.7%)	
Higher than before	54 (34.8%)	101 (65.2%)		41 (26.5%)	114 (73.5%)	
Daily exercise duration (min)			<0.001			0.009
<30	251 (49.9%)	252 (50.1%)		143 (28.4%)	360 (71.6%)	
30–60	172 (32.5%)	357 (67.5%)		111 (21.0%)	418 (79.0%)	
>60	42 (38.2%)	68 (61.8%)		21 (19.1%)	89 (80.9%)	

### 3.2 Multivariate logistic regression analysis of correlates of depression

Multivariate logistic regression was conducted to identify independent factors associated with depression, after controlling for potential confounders. Depression was defined as a CES-DC score >15, and non-depression as a score ≤15. Several variables were significantly associated with depression. Male participants had lower odds of depression compared to females (OR = 0.765, 95% CI: 0.588–0.995, P = 0.049). Adolescents who reported sleeping less than 6 hours per night were at significantly higher risk of depression than those with longer sleep durations (OR = 4.021, 95% CI: 2.152–7.534, P < 0.001). Reduced learning efficiency was also a strong correlate of depression. Participants who perceived their learning efficiency to be lower than before the pandemic had significantly increased odds of depression (OR = 2.853, 95% CI: 2.153–3.771, P < 0.001). In addition, adolescents who engaged in <30 minutes of daily physical activity had higher odds of depression compared to those with ≥30 minutes per day (OR = 1.722, 95% CI: 1.342–2.226, P < 0.001) (**Table 2**).

**Table 2:** Multivariate logistic regression analysis of correlates of depression.

Variables	Regression coefficient	Wald value	OR value	95% CI	P-value
Gender (male vs female)	-0.268	3.982	0.765	0.588 - 0.995	0.049
Sleep duration each day (<6h vs ≥6h)	1.392	18.946	4.021	2.152 - 7.534	<0.001
Learning efficiency (lower than before vs not lower than before)	1.048	53.992	2.853	2.153 - 3.771	<0.001
Physical exercise duration each day (<30 min vs ≥30 min)	0.543	17.878	1.722	1.342 - 2.226	<0.001

### 3.3 Multivariate logistic regression analysis of correlates of anxiety

Multivariate logistic regression was performed to identify independent correlates of anxiety, defined as a GAD-7 score ≥5. Scores <5 were categorized as non-anxiety. Grade level emerged as a significant predictor: junior secondary school students had lower odds of anxiety compared to senior high school students (OR = 0.438, 95% CI: 0.286–0.752, P = 0.005). School type approached statistical significance, with students from non-key schools showing a higher likelihood of anxiety than those attending key schools (OR = 0.711, 95% CI: 0.489–1.102, P = 0.063). Decreased learning efficiency was significantly associated with higher odds of anxiety (OR = 2.598, 95% CI: 1.695–3.387, P < 0.001). Physical activity was also a significant factor: adolescents engaging in <30 minutes of daily exercise had increased odds of anxiety compared to those with ≥30 minutes of activity (OR = 1.653, 95% CI: 1.299–2.521, P < 0.001) (**Table 3**).

**Table 3:** Multivariate logistic regression analysis of correlates of anxiety.

Variables	Regression coefficient	Wald value	OR value	95% CI	P-value
Grade (junior secondary school vs senior high school)	-0.826	7.298	0.438	0.286 - 0.752	0.005
School type (key school vs non-key school)	-0.341	3.358	0.711	0.489 - 1.102	0.063
Learning efficiency (lower than before vs not lower than before)	0.955	21.596	2.598	1.695 - 3.387	<0.001
Physical exercise duration each day (<30 min vs ≥30 min)	0.503	12.477	1.653	1.299 - 2.521	<0.001

## 4 Discussion

The findings of this study have important public health implications, particularly in the context of pandemic-related crises. The association between reduced PA and increased depression and anxiety among adolescents suggests the need for public health policies that prioritize accessible PA opportunities during periods of social distancing or lockdowns. Community programs should focus on promoting home-based or low-contact physical activities, such as virtual fitness classes, outdoor walking, or cycling initiatives, to maintain mental health in youth. Policymakers could also advocate for integrating PA into virtual education platforms to ensure continuous engagement in physical activity, even during school closures. By addressing PA as a critical aspect of adolescent well-being during public health emergencies, these strategies could help mitigate the long-term mental health consequences of pandemics. The findings highlight the protective role of PA in the context of a global health crisis, providing evidence that lower levels of PA are independently associated with higher levels of depression and anxiety among youth. The results of this study underscore the importance of promoting accessible forms of physical activity as a preventive and therapeutic strategy for mental health during periods of social isolation, such as pandemics. As adolescents struggle with school closures, reduced social interaction, and uncertainty, physical activity serves as a modifiable factor that can help buffer against the psychological strain of these stressors. Clinically, these findings advocate for targeted interventions aimed at increasing PA among adolescents, particularly in environments where access to traditional sports and physical education may be limited. This study contributes to the growing body of evidence that supports PA as a cost-effective, non-pharmacological intervention to support adolescent mental health, particularly during times of crisis.

The present study examines the relationship between physical activity (PA) and depressive and anxiety symptoms in adolescents during the COVID-19 pandemic. Our findings show that lower levels of daily physical activity were significantly associated with higher risks of both depression and anxiety. Specifically, adolescents engaging in less than 30 minutes of physical activity per day exhibited markedly higher odds of developing depressive (OR = 1.722) and anxiety symptoms (OR = 1.653), even after controlling for confounding factors. These results underscore the crucial role of PA as a protective factor against mental health challenges, especially during periods of significant stress such as the COVID-19 pandemic. Physiologically, regular PA has been shown to influence several biological systems linked to mental health (14, 15). Notably, PA modulates hypothalamic-pituitary-adrenal (HPA) axis function, enhances brain-derived neurotrophic factor (BDNF) expression, and reduces systemic inflammation—mechanisms that are all implicated in the pathophysiology of depression and anxiety (16, 17). Exercise has also been associated with positive neurochemical changes, particularly increased serotonergic and dopaminergic activity, which are essential for mood regulation and anxiety reduction. This finding aligns with existing literature, which suggests that exercise-induced neuroplasticity can help buffer against the psychological toll of stress (18, 19).

Psychosocially, PA offers additional benefits. Adolescents participating in structured or group-based exercises are more likely to experience social interaction, enhanced self-efficacy, and a sense of mastery, all of which mitigate stress and enhance emotional resilience. Adolescence is a critical period for the development of self-identity and emotional regulation, making the psychological benefits of PA particularly pronounced. Furthermore, our findings suggest that adolescents who maintain regular PA routines are more likely to exhibit healthier sleep hygiene and improved academic engagement—two factors strongly linked to lower levels of depression and anxiety. These results suggest that PA can indirectly benefit adolescent mental health by promoting overall well-being and stability in other areas of life. Our study’s findings are consistent with previous research conducted both before and during the COVID-19 pandemic. For instance, Bailey et al. (20) in their meta-analysis highlighted that physical activity interventions significantly reduced depressive symptoms in adolescents, demonstrating moderate effect sizes across randomized controlled trials. Similarly, a large-scale observational study in Norway found that lower levels of sport participation were associated with higher odds of depression and anxiety. During the pandemic, disruptions in daily routines, school closures, and reduced opportunities for outdoor activity exacerbated mental health issues in adolescents, as physical activity levels significantly declined. A study by Xiang et al. (21) found that adolescents who maintained moderate-to-vigorous PA during lockdown reported fewer depressive symptoms and better overall emotional well-being, echoing the findings of our study and underscoring the importance of preserving physical activity routines even during restrictive public health measures.

Our study also reinforces the concept of a dose-response relationship between PA and mental health. Global health authorities recommend at least 60 minutes of moderate-to-vigorous PA per day for adolescents, but our findings suggest that even modest reductions—specifically to less than 30 minutes—are associated with a significant increase in depression and anxiety symptoms. This finding is in line with previous studies that have suggested that the greatest protective effect occurs when adolescents transition from inactivity to regular movement, rather than simply accumulating higher levels of PA. Notably, sex- and age-specific patterns observed in our data also corroborate findings from other studies. Female adolescents consistently demonstrate higher rates of depression and anxiety, but studies, including one by Ekelund et al. (22), suggest that girls may experience equal or even greater mental health benefits from PA than boys, potentially due to differences in psychosocial stress reactivity or sociocultural factors. Similarly, our data show that older adolescents (ages 15–18) had higher rates of depression and anxiety, which may be linked to the increased academic and emotional pressures characteristic of this developmental stage. These findings support the notion that PA can be particularly beneficial during periods of heightened psychological stress, as seen in late adolescence. While some studies have focused on the role of organized sports clubs in protecting adolescent mental health, our study highlights the broader value of general physical activity, regardless of its structured form. Our results contribute to the growing body of evidence that emphasizes the positive effects of daily PA on adolescent mental health, irrespective of its specific context or organization. This broader perspective is essential for public health strategies aimed at promoting physical activity as a tool for improving mental health outcomes in adolescents, especially during periods of crisis such as the COVID-19 pandemic (23, 24).

These findings highlight the importance of clinicians, educators, and public health professionals actively promoting physical activity as part of adolescent mental health strategies. Screening for physical inactivity should be integrated into routine mental health assessments, with personalized recommendations for physical activity—tailored to individual capacities and preferences—considered as first-line preventive and adjunctive interventions for adolescents exhibiting symptoms of depression or anxiety. In particular, in post-pandemic contexts, schools and families should ensure that adolescents have regular access to safe and supportive environments for physical activity. This study has several strengths. First, it utilizes a large sample of over 1,100 adolescents, which enhances the statistical power and generalizability of the findings. Second, validated assessment tools, the CES-DC for depressive symptoms and the GAD-7 for anxiety—were used, ensuring reliable measurement of mental health status. Third, the study employed multivariate logistic regression analyses to adjust for key confounders, such as gender, age, learning efficiency, and sleep duration, thereby isolating the independent association between physical activity and mental health outcomes.

Several limitations should be acknowledged. First, the cross-sectional design limits causal inference; while significant associations were observed, it remains unclear whether low PA contributes to depression and anxiety, or whether these symptoms reduce motivation for PA. Longitudinal studies with repeated measures of PA, mental health symptoms, sleep duration, academic performance, and social engagement are needed to clarify temporal relationships. Second, PA was assessed via a single self-reported item without capturing frequency, intensity, or activity type, which may lead to exposure misclassification due to recall or social desirability bias. Future research should incorporate validated multi-item instruments or objective measures to improve measurement accuracy. Third, mental health outcomes were identified based on validated symptom thresholds rather than clinical diagnosis, which may overestimate prevalence but are appropriate for population-level screening. Fourth, the web-based survey design may introduce sampling bias, as adolescents with limited internet access or greater concern about mental health may have been overrepresented. Additionally, the study was geographically confined to Pidu District, Chengdu City in China—limiting generalizability to rural or socioeconomically diverse populations. Future studies should include more representative samples across different settings. Lastly, future research should evaluate scalable interventions—such as virtual fitness programs, outdoor activity initiatives, and integration of PA into online or hybrid school curricula—to enhance adolescent PA engagement.

## 5 Conclusions

In conclusion, this study identifies limited physical activity, specifically engaging in less than 30 minutes of daily exercise, as a significant independent risk factor for depressive symptoms in adolescents. Alongside female gender, reduced sleep duration, and decreased learning efficiency, insufficient physical activity emerged as a key modifiable contributor. These findings emphasize the critical role of promoting regular physical exercise in preventing adolescent depression.

## Data Availability

The original contributions presented in the study are included in the article/supplementary material. Further inquiries can be directed to the corresponding author.
